# Prevalence and risk of adverse intrapartum-related outcomes in Uganda: a cross-sectional study with nested case–control

**DOI:** 10.1136/bmjopen-2025-099256

**Published:** 2025-10-14

**Authors:** Phillip Wanduru, Manuela Straneo, Samantha Sadoo, Cally J Tann, Angelina Mwesige Kakooza, Rolland Mutumba, Kristi Sidney Annerstedt, Peter Waiswa, Claudia Hanson

**Affiliations:** 1 Department of Global Public Health, Karolinska Institute, Stockholm, Stockholm County, Sweden; 2 Department of Health Policy Planing and Managment, Makerere University School of Public Health, Kampala, Central Region, Uganda; 3 London School of Hygiene & Tropical Medicine, London, UK; 4 Department of Pediatrics and Child Health, Makerere University, Kampala, Uganda; 5 Iganga Hospital, Iganga, Uganda

**Keywords:** OBSTETRICS, Public health, Epidemiology

## Abstract

**Abstract:**

**Introduction:**

Intrapartum-related complications are a leading cause of adverse perinatal outcomes, including stillbirths, neonatal deaths and intrapartum-related neonatal encephalopathy (IP-NE). We assessed the prevalence of adverse intrapartum-related outcomes, evaluated the association between IP-NE and obstetric and fetal risk factors, and examined whether emergency referral and emergency caesarean section (CS) modified this association through interaction effects.

**Design:**

Cross-sectional with a nested case–control study.

**Setting:**

Two hospitals in rural Eastern Uganda.

**Population:**

Women giving birth to a live or stillborn baby weighing >2000 g between June and December 2022.

**Methods:**

We used prospectively collected perinatal e-registry data to assess the prevalence of adverse perinatal outcomes. Logistic regression with interaction with postregression margins analysis was used to determine the association between IP-NE and emergency referral and emergency CS across risk groups of hypertensive disorders, antepartum haemorrhage, prolonged/obstructed labour and birth weight.

**Main outcome measures:**

Adverse perinatal outcomes were stillbirths, 24-hour neonatal deaths and IP-NE (defined as Apgar score <7 at 5 min, cord blood lactate ≥5.5 mmol/L and Thompson score ≥5).

**Results:**

Of 6550 births, 10.2% had an adverse perinatal outcome: 3.8% stillbirths, 0.6% neonatal deaths and 5.7% IP-NE. Adverse outcomes were higher among neonates whose mothers had antepartum haemorrhage (31.3%) or prolonged/obstructed labour (27.2%) compared with those whose mothers had no complications. Emergency referral and CS did not change the association between IP-NE and obstetric risk, except in prolonged/obstructed labour. Without emergency CS, the predicted probability of IP-NE was 0.73 (95% CI 0.51 to 0.95); with CS, it decreased to 0.45 (95% CI 0.39 to 0.50).

**Conclusions:**

Neonates born to mothers with obstetric complications had low healthy survival rates. Emergency referral and CS did not alter the risks of IP-NE in women with obstetric complications except for obstructed or prolonged labour, highlighting that these interventions may not be implemented with sufficient timeliness or quality, and/or that additional, more targeted strategies beyond referral and CS are needed to address IP-NE.

**STRENGTHS AND LIMITATIONS OF THIS STUDY**

- Large, prospectively collected cohort of 6550 births from two high-volume maternity units in Uganda, with 99% data completeness.
- Standardised follow-up of perinatal outcomes, including rigorous assessment of intrapartum-related neonatal encephalopathy (IP-NE) with cord blood lactate and neurological evaluation.
- State-of-the-art diagnosis of IP-NE requires assessment of umbilical artery pH and base deficit; however, this is not feasible in rural public hospital settings, where reliance is instead placed on umbilical lactate.
- Stillbirths were classified as antepartum or intrapartum based on maceration, a method known to be unreliable.

## Introduction

 Intrapartum-related complications are a leading cause of neonatal deaths globally, accounting for over half a million deaths annually.^1^ Half of these deaths occur in sub-Saharan Africa (SSA).^1^ Survivors may develop neurological injuries with lifelong sequelae.^2^ Globally, intrapartum-related neonatal encephalopathy (IP-NE) is the second leading cause of age-standardised disability-adjusted life-years among neurological disorders.^3^

Efforts to prevent intrapartum-related complications have focused on facility births, and major increases in these have been seen in the last decade; in Uganda, over 90% of births now occur in facilities.^4^ However, this shift has not been paralleled by a corresponding reduction in adverse perinatal outcomes, which the WHO largely attributes to inadequate quality of intrapartum care.^5 6^ In response, WHO guidelines and key Ugandan policies prioritise improving the quality of care in health facilities.^5 7 8^

Guidelines and policies in SSA do not sufficiently acknowledge the interrelatedness and complexities of intrapartum-related complications. Cohort studies crossing the obstetric/paediatric divide are rare in low-income and middle-income countries. Most studies report a single adverse perinatal outcome within their domain, while few studies examining obstetric factors have attempted to include outcomes of IP-NE.^9^,^12^

In addition, there is a lack of evidence on the effectiveness of emergency interventions for preventing IP-NE from SSA. Risk factors for IP-NE, such as hypertensive disorders,^13 14^ obstructed or prolonged labour,^11 15^ antepartum haemorrhage,^11 13^ maternal infections^16^ and low birthweight births,^14 15^ are well established in literature. However, the effectiveness of key emergency interventions, including emergency caesarean section (CS) and emergency referral, in modifying the association between these risk factors and IP-NE remains underexplored. Although widely recognised as critical strategies to reduce IP-NE, to our knowledge, no studies in SSA have examined whether these emergency interventions modify the association between these risk factors and IP-NE risk. Most analyses treat these emergency interventions as risk factors rather than effect modifiers, which can misrepresent their role and limit understanding of their true impact in reducing IP-NE among high-risk births.

Here, we assessed the prevalence of adverse perinatal outcomes (stillbirths, 24-hour neonatal deaths and IP-NE) by obstetric and fetal risk factors at two hospitals in Eastern Uganda. Second, we examined whether emergency referral and emergency CS modified the association between IP-NE and obstetric or fetal risk factors.

## Methods

### Study design

This was a cross-sectional study with a nested case–control. Our cross-sectional study used prospectively collected data derived from the Action Leverage Evidence to Reduce Perinatal Mortality and Morbidity (ALERT) e-registry, as described in the study protocol,^17^ which included a 24-hour follow-up of all births to identify adverse perinatal outcomes. A nested case–control study examined the association between IP-NE and obstetric and fetal risk factors and assessed whether emergency referral and emergency CS modified this association through interaction effects.

### Study setting

The study was conducted in a Regional Referral Hospital and a General Hospital in Eastern Uganda, serving a predominantly rural population of about 4 million. Uganda has 14 regions, each with a tiered referral health system, with Regional Referral Hospitals at the highest level, followed by General Hospitals that typically serve multiple districts. Health Centre IVs and IIIs also provide maternity care and handle most uncomplicated obstetric cases within these districts. Both hospital types provide maternity care—including CSs, neonatal care, blood transfusions and laboratory services. Neonatal care units at these facilities offer WHO level 2 care.^18^ Regional Referral Hospitals are staffed by consultant obstetricians and paediatricians, while General Hospitals have non-consultant specialists. The CSs are available 24/7, though delays occur due to stockouts and staff shortages. These public hospitals offer free services and handle high birth volumes, with approximately 7000 deliveries annually each. Despite service availability, staffing remains limited and several studies have documented quality-of-care gaps.^1719^,^21^

### Study population and data collection

The ALERT perinatal e-registry collected data on risks, intrapartum care and outcomes of all mother–baby pairs admitted to the study sites, abstracted from standardised paper-based maternity charts and uploaded onto the REDCap platform.^17 21^ For the cross-sectional study, we extracted data of births that occurred between 1 June 2022 and 31 December 2022, excluding neonates weighing less than 2000 g. We excluded neonates weighing <2000 g because physiological immaturity determines outcomes through distinct pathways, such as intraventricular haemorrhage and respiratory distress.^22^,^26^ Lower Apgar scores tend to indicate prematurity rather than intrapartum-related complications, making our screening for IP-NE, our primary study outcome, inappropriate.^27^ We also excluded neonates with congenital abnormalities.

In the nested case–control study, cases were neonates who survived 24 hours with IP-NE defined by Apgar <7 at 5 min, and cord blood lactate ≥5.5 mmol/L, and a Thompson score ≥5.^28^ For each case, four healthy neonates were consecutively recruited as controls, defined as being born alive and in good condition with an Apgar score >7 at 5 min. Specifically for the case–control study, we supplemented the data with maternal interviews conducted by study staff to gather sociodemographic variables, including maternal employment, marital status, level of education and distance travelled from the village to the hospital.

### Study variables

#### Outcome variables

For the cross-sectional study, we defined three mutually exclusive primary outcomes, including (a) Stillbirths, defined as a fetus delivered without signs of life, as observed and documented by health workers present at birth. If evidence of maceration was present, they were classified as ‘antepartum-related stillbirth’; those without evidence of maceration were classified as ‘intrapartum-related stillbirths’; (b) neonatal deaths, defined as death of a neonate within 24 hours of birth regardless of whether signs and symptoms of IP-NE were observed; and (c) IP-NE neonates who met the criteria for IP-NE and were alive at 24 hours (see definition below).

In the case–control study, the primary outcome of interest was IP-NE among neonates alive at 24 hours. The procedure for determining IP-NE cases involved drawing a small cord blood sample from the umbilical artery through a needle prick from neonates with an Apgar score <7 at 5 min. We used the ‘Nova’ lactate point-of-care metre,^29^ as advanced laboratory options to assess pH or base deficits were unavailable in our setting. All neonates with cord blood lactate value ≥5.5 mmol/L^30^ were further evaluated for signs of encephalopathy using the Thompson score by trained nurses supervised by a paediatrician.^31^ The score was developed and validated in a SSA setting and consists of nine items, including tone, level of consciousness, clinical fits, posture, Moro reflex, grasp reflex, sucking reflex, respiration and fontanelle.^31^ The total score ranges from 0 to 22; more severely affected neonates have a higher score.^31 32^ The Thompson score examination was performed twice, 6–7 hours and 12–13 hours after birth.^31^

The traditional Thompson score categorises a range of 1–10 as mild, 11–14 as moderate and 15–22 as severe.^32^ However, Horn *et al* demonstrated that low Thompson scores (1–4) would likely have a normal electroencephalogram.^33^ Thus, to improve diagnostic precision, we adjusted categories as follows: a Thompson score of 5–10 is now considered mild, 11–14 is considered moderate and 15–22 is considered severe in cases of IP-NE.

#### Independent variables

These variables comprised obstetric and fetal risk factors. Obstetric risk factors included parity (categorised as primipara, 1–4 pregnancies or ≥5 pregnancies) and extra-uterine infections, which were defined as the presence of HIV, syphilis or malaria as documented in the patient charts. Furthermore, we included hypertensive disorders—defined as pre-eclampsia, eclampsia, gestational hypertension or chronic hypertension—along with antepartum haemorrhage and prolonged or obstructed labour. Prolonged or obstructed labour was defined as: (1) labour lasting more than 18 hours in cases of fetal vertex presentation or (2) malpresentation or other conditions that precluded spontaneous vaginal birth. The fetal risk factors included the sex of the neonate, either male or female, and birth weight, classified as low birth weight (<2500 g), normal birth weight (2500–4000 g) and high birth weight (>4000 g).^34^

#### Effect modifiers

In the case–control study, we examined two primary effect modifiers—emergency referral and emergency CS—as documented in patient records. Emergency referral was defined as transferring a mother from another facility after the onset of labour. Emergency CS referred to cases where the decision to perform a CS was made after the onset of labour, excluding elective CS. We assessed how these interventions modified the association between obstetric and fetal risk factors (hypertensive disorders, antepartum haemorrhage, prolonged or obstructed labour and birth weight) and IP-NE.

### Data analysis

We used Stata V.16 (Stata) for all analyses. In the cross-sectional study, we calculated descriptive statistics of frequencies with 95% CIs stratified by selected obstetric and fetal risk factors. Our power calculation, conducted post hoc, confirmed that the case–control study was adequately powered (online supplemental figure 1). We used χ^2^ and Fisher’s exact tests to determine whether differences in obstetric and fetal characteristics between cases and controls were statistically significant. Further, we used multivariable logistic regression to assess whether emergency referral and emergency CS modified the association between obstetric and fetal risk factors and IP-NE. As shown in online supplemental figure 2, each model included an interaction term between the presence or absence of intervention and a specific risk factor, adjusting for relevant confounders.

Effect modification was computed using postestimation margins analysis, which calculates the predicted probability of IP-NE for all combinations of intervention status (present or absent) and risk factor categories. This method uses the regression coefficients, including interaction terms, to estimate adjusted probabilities reflecting how the intervention modifies the effect of each risk factor on IP-NE risk. The analysis was applied to all eight combinations arising from the two levels of each intervention crossed with the four key risk factors, allowing comprehensive assessment of modification effects across relevant strata.

### Confounding

In each of the regression models, we adjusted for sociodemographic factors, including maternal level of education, maternal age, maternal marital status and employment status, as these are indicators of maternal economic well-being and have a recognised association with increased risk of IP-NE.^15 35 36^ These sociodemographic factors are also associated with obstetric risk factors, including hypertensive disorders,^37^ antepartum haemorrhage,^11^ obstructed labour, prolonged labour^15 36 38^ and low birthweight neonates.^15 39^ We, therefore, viewed them as essential confounders to adjust for in all models. In addition, we adjusted for distance, a common barrier to accessing timely care in this setting,^40^ and parity since this is a known confounder associated with IP-NE and obstetric complications.^11^ We adjusted for emergency CS and vice versa for models examining emergency referrals to isolate their effects.

### Patient and public involvement

It was not appropriate or possible to involve patients or the public in the design, conduct, reporting or dissemination plans of this study

## Results

**Figure 1:**
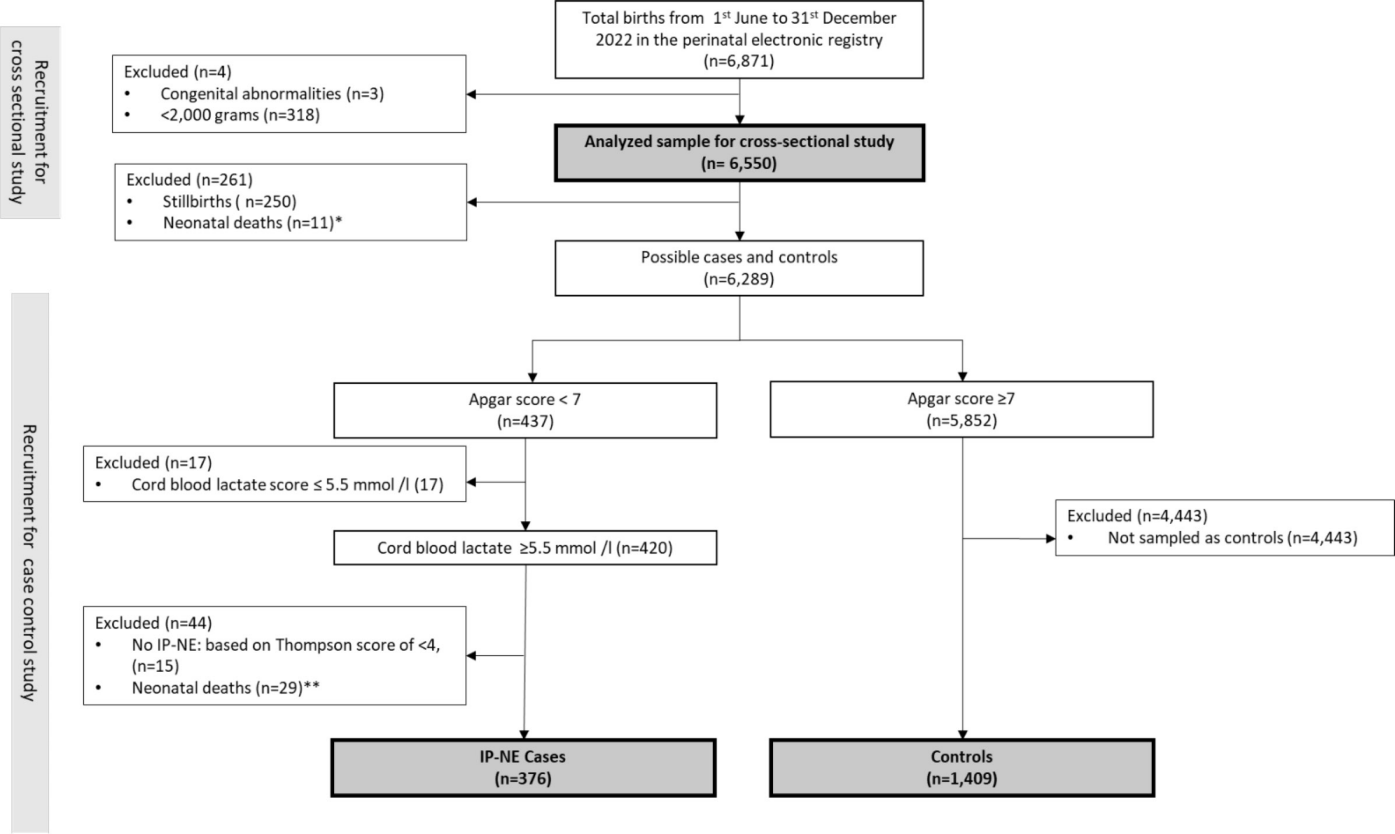
The recruitment process for study participants for cross-sectional and case–control study arms (1 June 2022–31 December 2022). (A) Neonatal deaths*: deaths within 1 hour after birth, (**B**) Neonatal deaths**: deaths >1 hour but within 24 hours.

Among the 6871 births recorded between 1 June 2022 and 31 December 2022 (figure 1), 6550 met the inclusion criteria, with 318 excluded due to weighing <2000 g and 3 had congenital abnormalities. The case–control analysis comprised 1785 neonates, 376 cases and 1409 controls.

Table 1 displays maternal characteristics and birth outcomes for the total cohort. One-sixth (16.6%) of mothers giving birth were under 20 years of age, while 9% were over 35. One-third (34.8%) of all births were primiparous mothers, while 15.6% were grand multipara (5+deliveries). The CS rate was 27.3%, of which the majority were emergency CS (18.3% compared with 9.0% elective CS, such as CS for a previous scar). Neonates with low birth weight were 12.6%, 83.3% were between normal birth weight and 4.1% were high birth weight. Of 6550 births, 10.2% resulted in adverse perinatal outcomes. This included 3.7% stillbirths (1.2% antepartum-related events and 2.6% intrapartum-related events). Neonatal deaths within 24 hours were 0.6% of all births, and IP-NE was diagnosed in 5.7%.

**Table 1:** Maternal and obstetric characteristics and 24-hour birth outcomes (1 June 2022–31 December 2022; n=6550 births)

	Cross-sectional study Births (N=6550)		Case–control study Births (n=1785) n(%)				
	n (%)		Controls N=1409		Cases n=376		P value
**Maternal and obstetric characteristics**							
Maternal age							
<20	1089	(16.6)	218	(15.5)	83	(22.1)	0.01
20–24	2254	(34.4)	488	(34.7)	133	(35.5)	
25–29	1653	(25.2)	359	(25.5)	88	(23.5)	
30–34	970	(14.8)	238	(16.9)	52	(13.9)	
35+	584	(8.9)	106	(7.5)	19	(5.1)	
Maternal employment status							
Employed	2620	(40.0)	607	(43.1)	117	(31.1)	<0.001
Non-employed	3930	(60.0)	802	(56.9)	259	(68.9)	
Parity							
Primipara	2277	(34.8)	464	(32.9)	175	(46.8)	<0.001
1–4	3250	(49.6)	731	(51.9)	146	(38.9)	
5+	1022	(15.6)	213	(15.1)	54	(14.4)	
Emergency referral							
Yes	665	(10.2)	202	(14.3)	101	(26.9)	<0.001
No	5910	(89.8)	1207	(85.7)	275	(73.1)	
Mode of birth							
Vaginal birth	4744	(72.4)	1089	(77.3)	231	(61.4)	<0.001
Emergency caesarean section	1197	(18.3)	249	(17.7)	135	(36.0)	
Elective caesarean section	590	(9.0)	71	(5.0)	5	(1.3)	
Assisted vaginal birth	19	(0.3)					
**Additional variables collected for case–control study**							
Maternal highest level of education*							
None/primary	–	–	372	(26.4)	95	(25.3)	0.01
Secondary level	–	–	840	(59.7)	260	(69.1)	
Tertiary level	–	–	197	(14.0)	21	(5.6)	
Maternal marital status							
Married/cohabiting	–	–	1267	(89.9)	251	(66.8)	<0.001
Not married/cohabiting	–	–	142	(10.1)	125	(33.2)	
Distance from facility							
Within 20 km radius	–	–	1163	(82.5)	252	(67.0)	<0.001
Beyond 20 km radius	–	–	246	(17.5)	124	(33.1)	
**Birth outcomes**							
Neonate sex							
Male	3368	(51.4)	687	(48.8)	222	(59.2)	<0.001
Female	3182	(48.6)	722	(51.2)	153	(40.8)	
birth weight							
Low birth weight (<2500 g)	823	(12.6)	72	(5.1)	46	(12.2)	<0.001
Normal birth weight (2500–4000 g)	5453	(83.3)	1337	(94.9)	330	(87.8)	
High birth weight (>4000 g)	267	(4.1)					
Birth outcomes							
Healthy neonates	5884	(89.8)	1409				
Intrapartum-related neonatal encephalopathy	376	(5.7)			376		
Antepartum-related stillbirths	80	(1.2)					
Intrapartum-related stillbirths	170	(2.6)					
Early neonatal deaths	40	(0.6)					

Missing: birth weight n=7, parity n=1.

* Maternal education, marital status and distance not collected in e-registry.

Table 2 shows the prevalence of obstetric and fetal risk factors by adverse perinatal outcomes. Among mothers who experienced antepartum haemorrhage, 31.3% had adverse perinatal outcomes, including 7.3% stillbirths and 22.9% IP-NE. Similarly, women experiencing prolonged or obstructed labour, 27.2% experienced adverse perinatal outcomes, including 6% stillbirths and 19.1% IP-NE. Among those with hypertensive disorders, 20.9% experienced adverse perinatal outcomes; 6.8% were stillbirths and 11.4% developed IP-NE. Online supplemental figure 3 shows the detailed outcomes of birth.

**Table 2:** Prevalence of 24-hour birth outcomes by obstetric and fetal risk groups at two hospitals in Eastern Uganda, 1 June 2022–31 December 2022 (n=6550 births)

Birth outcomes; N=6550		Total	Healthy neonates	Stillbirths		Neonatal Deaths N=40 n% (95% CI)	Intrapartum-related neonatal encephalopathy, N=376 n% (95% CI)
			N=5884 n% (95% CI)	Antepartum-related stillbirth, N=80 n% (95% CI)	Intrapartum-related stillbirth, N=170 n% (95% CI)		
**Obstetric risk factors**							
Parity	Primipara	2277	87.7 (86.3 to 89.0)	1.0 (0.6 to 1.5)	2.6 (2.0 to 3.4)	1.0 (0.6 to 1.5)	7.7 (6.6 to 8.9)
	1–4	3250	93.8 (92.9 to 94.6)	1.1 (0.8 to 1.5)	2.3 (1.8 to 2.9)	0.6 (0.3 to 0.9)	2.3 (1.8 to 2.9)
	5+	1022	86.5 (84.3 to 88.5)	2.2 (1.4 to 3.2)	3.5 (2.5 to 4.8)	0	7.8 (6.3 to 9.7)
Extrauterine infections	Yes	376	91.2 (87.9 to 93.9)	1.3 (0.4 to 3.1)	2.4 (1.1 to 4.5)	1.3 (0.4 to 3.1)	3.7 (2.1 to 6.2)
	No	6034	89.6 (88.8 to 90.3)	1.2 (1.0 to 1.5)	2.7 (2.3 to 3.1)	0.5 (0.4 to 0.8)	6.0 (5.4 to 6.6)
Hypertensive disorders	Yes	282	79.1 (73.9 to 83.7)	2.5 (1.0 to 5.0)	4.3 (2.2 to 7.3)	2.8 (1.2 to 5.6)	11.4 (7.9 to 15.6)
	No	6268	90.0 (89.2 to 90.7)	1.2 (0.9 to 1.5)	2.5 (2.1 to 2.9)	0.5 (0.3 to 0.7)	5.8 (5.2 to 6.4)
Antepartum haemorrhage	Yes	301	68.7 (63.2 to 74.0)	2.3 (0.9 to 4.7)	5.6 (3.3 to 8.9)	0.3 (0.1 to 1.8)	22.9 (18.3 to 28.1)
	No	6249	93.7 (93.0 to 94.2)	1.1 (0.9 to 1.5)	2.4 (2.0 to 2.9)	0.6 (0.4 to 0.8)	4.8 (4.4 to 5.4)
Prolonged or obstructed labour	Yes	533	72.8 (68.8 to 76.5)	1.1 (0.4 to 2.4)	4.9 (3.2 to 7.1)	2.1 (1.0 to 3.7)	19.1 (15.9 to 22.7)
	No	6017	91.3 (90.6 to 92.0)	1.2 (1.0 to 1.5)	2.3 (2.0 to 2.8)	0.5 (0.3 to 0.7)	4.6 (4.4 to 5.1)
**Fetal risk factors**							
Birth weight	Low birth weight	823	84.2 (81.5 to 86.6)	2.8 (1.8 to 4.2)	5.8 (4.3 to 7.7)	0.2 (0.03 to 0.9)	6.9 (5.3 to 8.9)
	Normal birth weight	5453	90.8 (90.0 to 91.2)	0.9 (0.7 to 1.1)	2.0 (1.7 to 2.4)	0.7 (0.5 to 0.9)	5.5 (4.9 to 6.1)
	High birth weight	267	88.0 (83.5 to 91.7)	3.0 (1.3 to 5.8)	4.5 (2.3 to 7.7)	0.8 (0.1 to 2.7)	3.6 (1.8 to 6.8)
Sex	Female	3182	90.9 (89.9 to 91.9)	1.2 (0.9 to 1.6)	2.7 (2.2 to 3.3)	0.6 (0.4 to 1.0)	6.6 (5.8 to 7.5)
	Male	3368	88.6 (87.4 to 89.7)	1.3 (0.9 to 1.7)	2.5 (2.0 to 3.1)	0.6 (0.4 to 0.9)	7.0 (6.2 to 8.0)
Overall		6550	89.8 (89.1 to 90.6)	1.2 (1.0 to 1.5)	2.6 (2.2 to 3.0)	0.6 (0.4 to 0.8)	5.7 (5.2 to 6.3)

Missing: birth weight n=7, parity n=1, extrauterine infections n=140; Extrauterine infections include HIV, malaria and syphilis.

**Figure 2:**
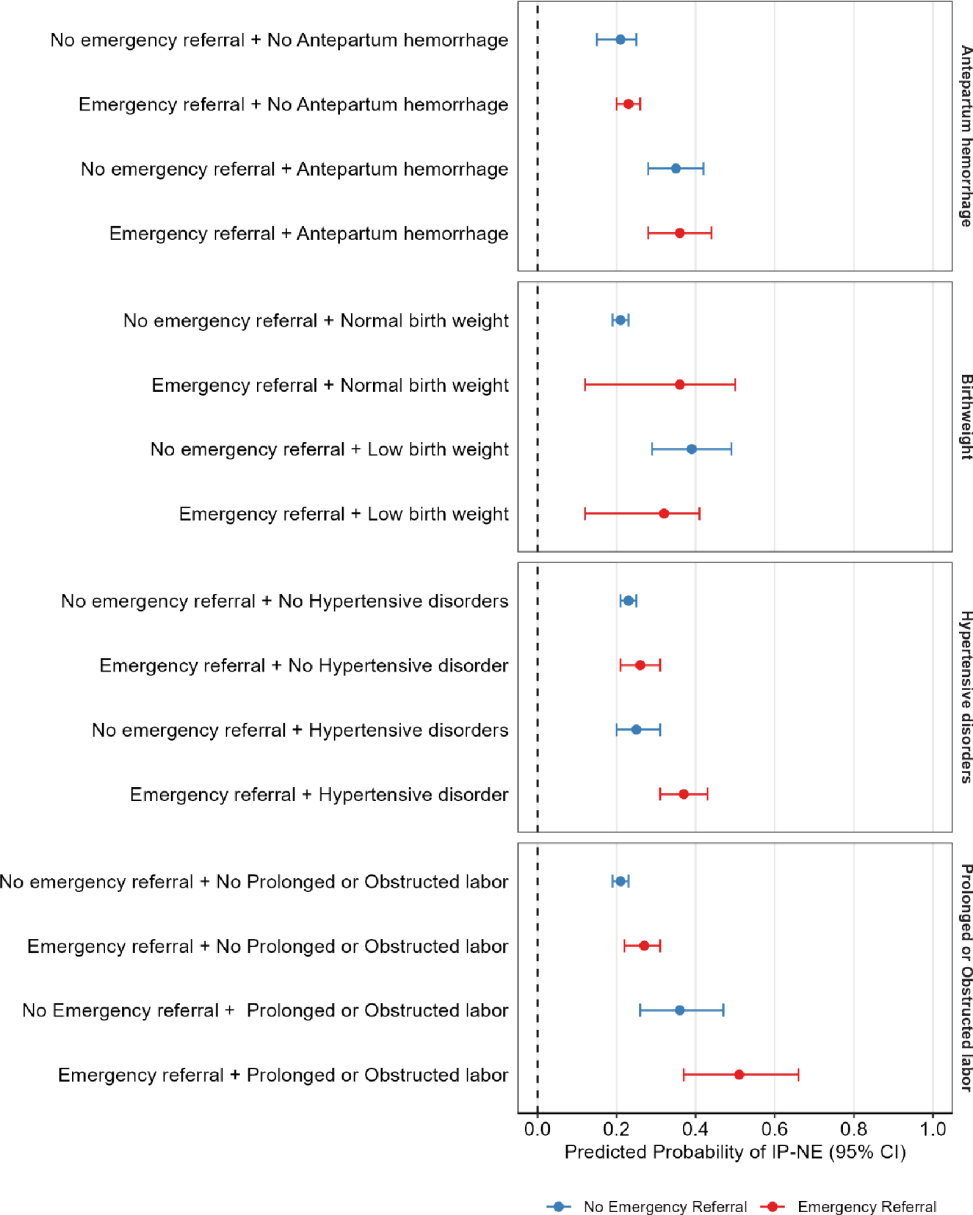
Predicted probability of IP-NE by obstetric risk groups and emergency referral using logistic regression margins. *(A) Birth weight we adjusted for maternal employment, marital status, maternal education, parity, antepartum haemorrhage, prolonged or obstructed labour, hypertensive disorders, distance to facility and emergency caesarean section. (**B**) Prolonged or obstructed labour adjusted for maternal employment, marital status, maternal education, parity, antepartum haemorrhage, birth weight, hypertensive disorder, distance to facility and emergency caesarean section. (**C**) Antepartum haemorrhage adjusted for maternal employment, marital status, maternal highest level of education, parity, prolonged or obstructed labour, birth weight, hypertensive disorders and distance to facility, emergency caesarean section. (**D**) Hypertensive disorder adjusted for maternal employment, marital status, maternal education, parity, antepartum haemorrhage, birth weight, prolonged or obstructed labour, distance to facility and emergency caesarean section. IP-NE, intrapartum-related neonatal encephalopathy.

In the case–control study, we found that risk groups—including low birthweight neonates, those whose mothers experienced prolonged or obstructed labour, antepartum haemorrhage and hypertensive disorders—had a higher predicted probability of IP-NE compared with those without these conditions, regardless of emergency referral status. For instance, among neonates whose mothers received emergency referral during labour, those with prolonged or obstructed labour had a predicted risk of IP-NE of 0.51 (95% CI 0.37 to 0.66), while those without prolonged labour had a lower predicted risk of 0.27 (95% CI 0.22 to 0.31). We also observed that emergency referral was associated with an increased predicted probability of IP-NE within the obstetric and fetal risk groups; however, these associations did not reach statistical significance. For example, neonates whose mothers experienced prolonged or obstructed labour and were not referred had a predicted risk of 0.36 (95% CI 0.26 to 0.47), which was higher but not statistically different from the risk of 0.51 (95% CI 0.37 to 0.66) in those who were referred (figure 2). Full details of predicted probabilities by obstetric risk group and emergency referral are provided in online supplemental table 1.

**Figure 3:**
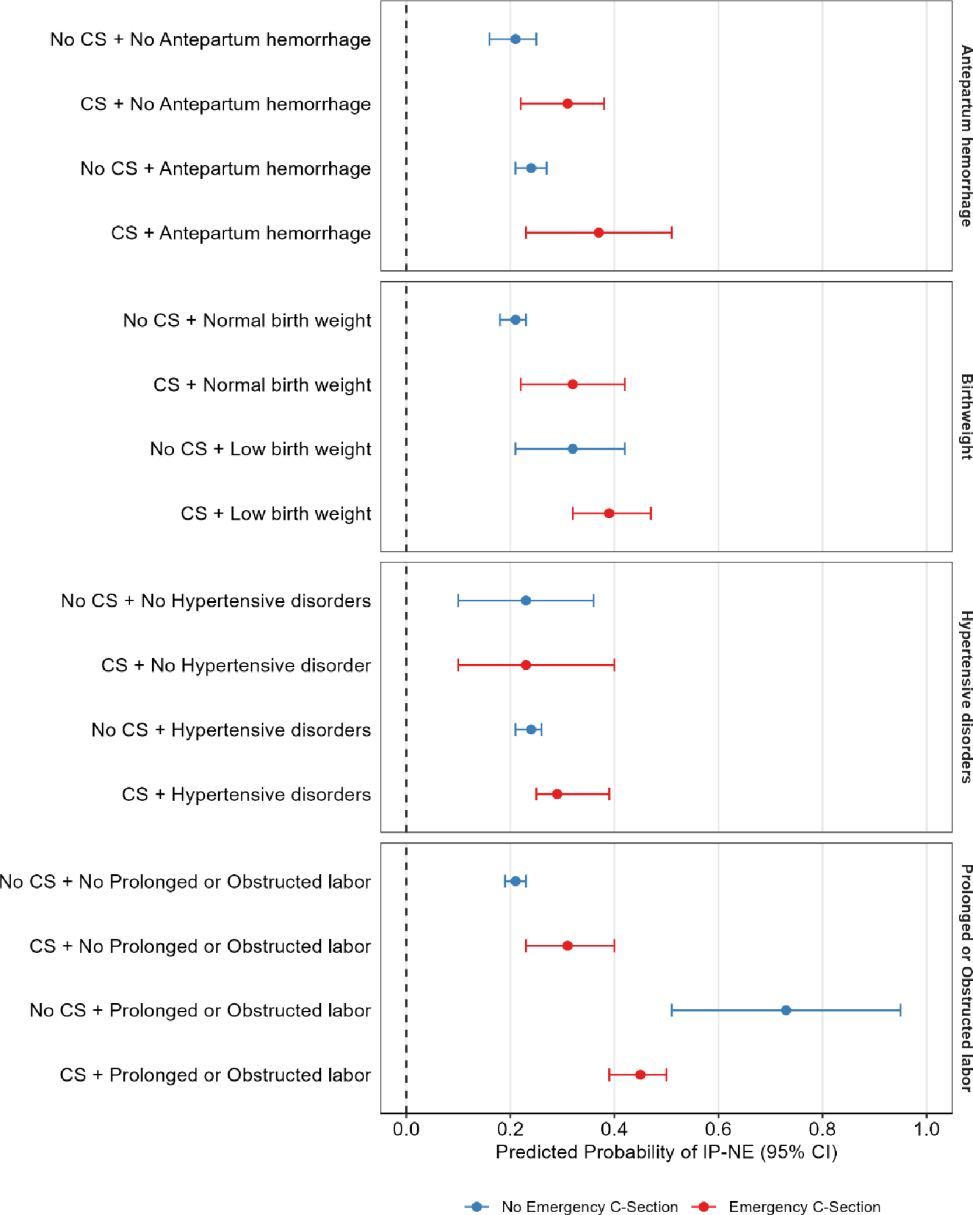
Predicted Probability of IP-NE obstetric risk groups and emergency caesarean section using logistic regression margins. *(A) Birth weight adjusted for maternal employment, marital status, maternal education, parity, antepartum haemorrhage, prolonged or obstructed labour, hypertensive disorders, distance to facility and emergency referral. (**B**) Prolonged or obstructed labour adjusted for maternal employment, marital status, maternal education, parity, antepartum haemorrhage, birth weight, hypertensive disorder, distance to facility and emergency referral. (**C**) Antepartum haemorrhage adjusted for maternal employment, marital status, maternal highest level of education, parity, prolonged or obstructed labour, birth weight, hypertensive disorders and distance to facility and emergency referral. (**D**) Hypertensive disorder adjusted for maternal employment, marital status, maternal education, parity, antepartum haemorrhage, birth weight, prolonged or obstructed labour, distance to facility and emergency referral. IP-NE, intrapartum-related neonatal encephalopathy.

Emergency CS was associated with an increased predicted probability of IP-NE within specific risk groups, but this was significant only among those with prolonged or obstructed labour. Neonates whose mothers experienced prolonged or obstructed labour and did not undergo emergency CS had the highest risk, at 0.73 (95% CI 0.51 to 0.95). In contrast, the risk was lower for those whose mothers with prolonged or obstructed labour did receive emergency CS, at 0.45 (95% CI 0.39 to 0.50) (figure 3). Full details of predicted probabilities by obstetric risk group and emergency CS are provided in the online supplemental table 2.

## Discussion

In this cross-sectional study with a nested case–control design, out of 6550 births in two high-volume Ugandan hospitals, we observed that 10.2% of births had adverse perinatal outcomes; 3.8% were stillbirths, 0.6% early neonatal deaths and 5.7% of neonates had an IP-NE. Adverse perinatal outcomes were highest among births complicated by antepartum haemorrhage (31.3%), followed by prolonged or obstructed labour (27.2%) and hypertensive disorders (20.9%), we found that emergency referral or undergoing an emergency CS did not significantly alter the risk of IP-NE across different obstetric and fetal risk groups, except for not having an emergency CS in prolonged or obstructed labour instances.

Our estimate of 3.7% of stillbirths concurs with other studies from Uganda’s stillbirth rates of 3.5%–5%.^16 21 41 42^ We observed a higher portion of intrapartum-related stillbirths (2.6%) compared with those that are antepartum-related (1.1%). Intrapartum-related stillbirths are associated with obstetric emergencies, whereas antepartum-related stillbirths are linked to maternal infections and fetal growth restriction.^43 44^ Consequently, a higher proportion of intrapartum-related stillbirths, compared with antepartum-related stillbirths, indicates a more significant issue regarding access to quality obstetric care in the population.

Regarding the burden of IP-NE, we found a prevalence of 5.7%. This prevalence varied across studies, with reports of 1% in a South African study,^45^ 10.6% in a Tanzanian study^10^ and 3% in a Ugandan study.^11^ Such variations may be attributed to differences in healthcare resources and access to care across these settings, even within the same region.

Our research indicates that neonates of mothers who experience complications such as antepartum haemorrhage, prolonged or obstructed labour, hypertensive disorders and those who were low birth weight have a higher rate of adverse perinatal outcomes. This was unsurprising, as it aligns with previous studies showing a heightened risk of IP-NE among neonates born to mothers with these complications.^11 16 46^ However, the absolute risk is striking.

Further, except for those with prolonged or obstructed labour, we did not observe a significant effect of emergency referral or CS on IP-NE across most obstetric and fetal risk groups. The risk of IP-NE was highest among neonates whose mothers experienced prolonged or obstructed labour and did not undergo an emergency CS, the appropriate intervention in such situations. In the case of antepartum haemorrhage, hypertensive disorders or low birth weight, a CS may be needed, but not always. Thus, our analysis underscores that other interventions and procedures might be needed to reduce the adverse neonatal event burden from these complications. In addition, delays in accessing care, a phenomenon common in rural SSA, may explain some missing effects.^20 47 48^ The delays primarily stem from a lack of resources in the health system, such as a lack of personnel and supplies for conducting an emergency CS and a lack of means of a functional ambulance system for referral. Diagnostic challenges in the identification of fetal distress and signs of encephalopathy also contribute to delays.

The strength of this study is the large sample of 6550 births from two sites, with data collected prospectively, achieving 99% data completeness. We present data on a range of key perinatal outcomes, capturing stillbirths, neonatal deaths and IP-NE, which was rigorously defined, including cord blood lactate measurement and standardised neurological assessment. Our study faced limitations. First, IP-NE is ideally diagnosed based on a fetal umbilical artery pH below 7.0 and/or a base deficit of ≥12 mmol/L, indicating significant fetal acid-base disturbances.^49^ Instead, we relied on a point-of-care NOVA lactate rapid test. Studies have indicated that lactate is an acceptable approximation when the capacity to test for acidaemia is unavailable.^50^,^52^ Our sample of 376 neonates with IP-NE in the case–control study was limited in size to assess predictors of IP-NE; this is why it is possible that we missed significant indicators. In addition, we categorised stillbirth into antepartum-related or intrapartum-related based on the presence or absence of maceration. However, using fetal appearance to determine the time of fetal death is not always reliable.^53^ A third of the births in the data set were missing information on the presence of a fetal heartbeat at admission, introducing a non-differential bias. Moreover, fetal heartbeat detection in the setting was measured solely using the Pinard stethoscope, which, based on experience and research, is unreliable in detecting fetal heartbeat.^54^

## Conclusions

Our study indicates that out of 6550 births in two high-volume public Ugandan hospitals, 1 in 10 neonates suffered an intrapartum-related adverse perinatal outcome. A staggering one-third of neonates whose birth is complicated by antepartum haemorrhage, prolonged or obstructed labour, and one-fifth of neonates whose birth is complicated by hypertensive disorders suffered adverse perinatal outcomes of stillbirth, early death and IP-NE. These findings highlight the urgent need for intervention. Emergency referral and CS demonstrated limited benefits in reducing IP-NE risk. Our study was not designed to provide reasons why emergency referral and CS did not alter the risk of IP-NE in a significant way; we believe that more work is needed to understand which interventions and intervention packages may have the largest effect on preventing adverse events. As we cannot rule out that delays in reaching definite care were an additional underlying reason, we suggest a focus on designing programme strategies that enhance access to obstetric emergency care services, such as emergency referrals and CS for those in need. Particular attention should be paid to rural populations facing significant financial and geographical barriers. Such strategies could include increasing rural public hospitals’ capacity for timely emergency caesarean deliveries by addressing human resource shortages and chronic supply deficiencies.

## Supplementary material

10.1136/bmjopen-2025-099256 online supplemental file 1

## Data Availability

Data are available on reasonable request.
