# Supplementary material for: Prevalence and risk of adverse intrapartum-related outcomes in Uganda: a cross-sectional study with nested case–control
Source: BMJ Open. 2025 Oct 14;15(10):e099256. doi: 10.1136/bmjopen-2025-099256 (PMC12519689; doi:10.1136/bmjopen-2025-099256)
Supplement: online supplemental file 1 [file bmjopen-15-10-s001.pdf]

Supplementary figures and tables

Supplementary Figure 1: Sample size power calculation for the case-control study (stata extract)

```
. power twoproportions 0.4615 0.5385, n1(376) n2(1409)

Estimated power for a two-sample proportions test
Pearson's chi-squared test
Ho: p2 = p1  versus  Ha: p2 != p1

Study parameters:

        alpha =    0.0500
            N =     1,785
           N1 =       376
           N2 =     1,409
        N2/N1 =    3.7473
        delta =    0.0770  (difference)
           p1 =    0.4615
           p2 =    0.5385

Estimated power:

        power =    0.7571
```

* To evaluate the statistical power of our case-control study, we used a two-sided confidence interval of 95%. The emergency CS rate (primary exposure) amongst cases was 53.85%, while the emergency CS rate among controls was 46.15%. With 376 cases and 1,409 controls, our analysis indicated a power of 75.7%, indicating that the study was adequately powered to detect significant associations.

Supplementary Figure 2: Models run in assessing the Association Between IP-NE and Selected Obstetric Risk Factors, Emergency Referral, and Emergency Cesarean Section

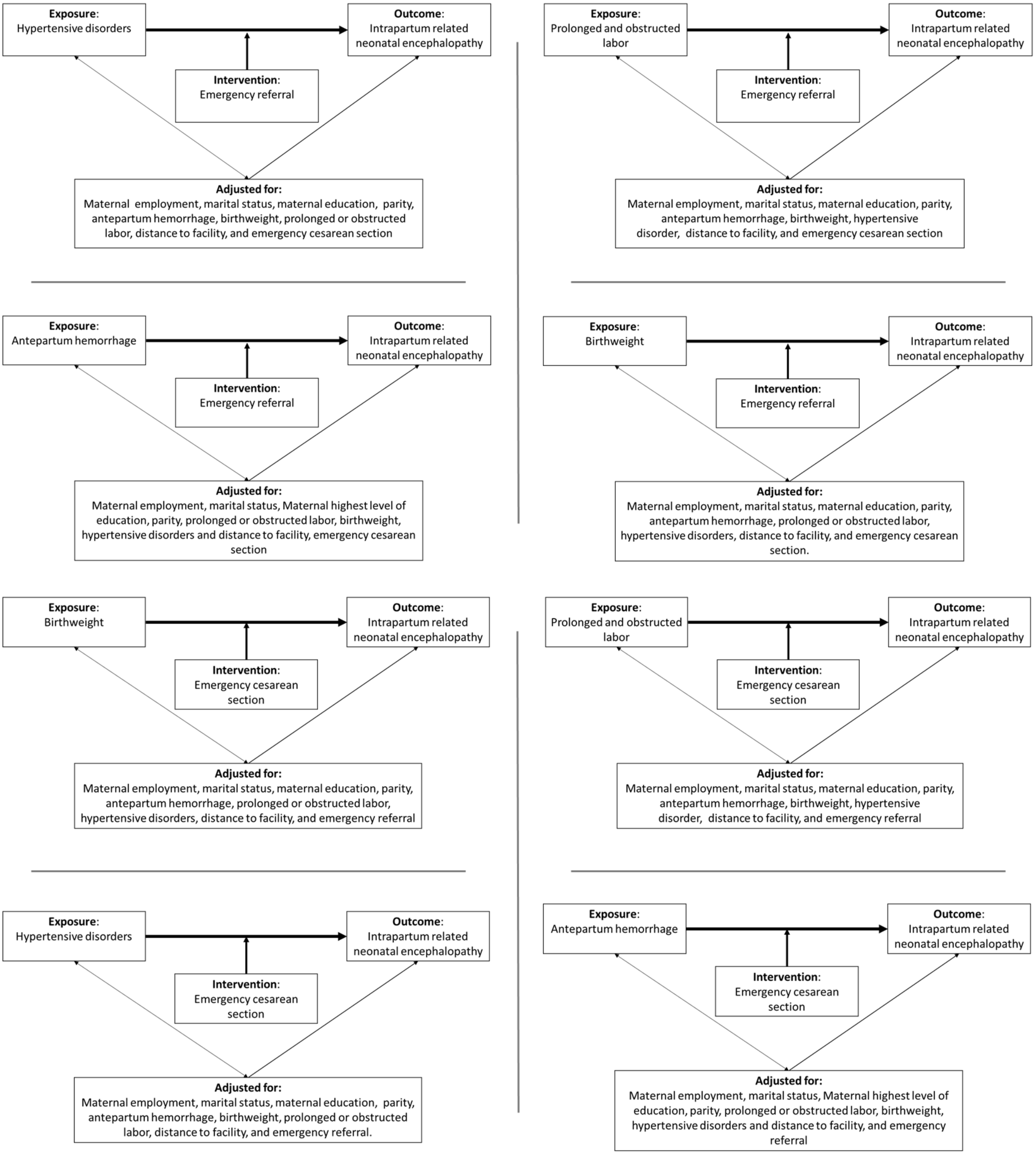

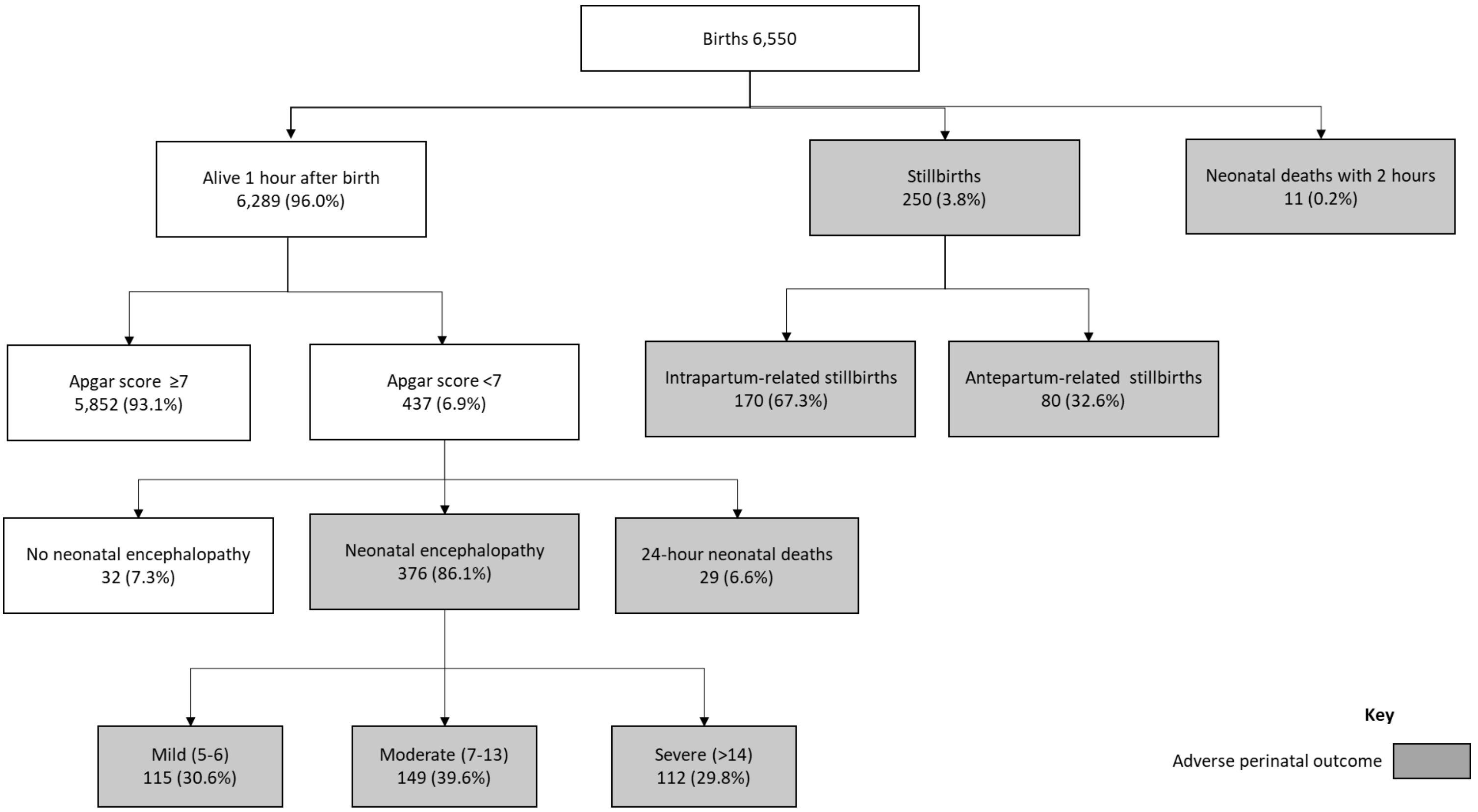

Supplementary figure 3: Outcomes of births conducted across two hospitals, from 1st June to 31st December 2022 (n=6,550 births).

Supplementary Table 1: Predicted Probability of IP-NE by Obstetric Risk Groups and Emergency Referral Using Logistic Regression Margins

**Obstetric and fetal risk**		Probability (95%CI)
Birthweight	No emergency referral + Normal birth weight	0.21 (0.19-0.23)
	Emergency referral + Normal birth weight	0.36 (0.12-0.50)
	No emergency referral + Low birth weight	0.39 (0.29-0.49)
	Emergency referral + Low birth weight	0.32 (0.12-0.41)
Prolonged or Obstructed labor	No emergency referral + No Prolonged or Obstructed labor	0.21 (0.19-0.23)
	Emergency referral + No Prolonged or Obstructed labor	0.27 (0.22-0.31)
	No Emergency referral + Prolonged or Obstructed labor	0.36 (0.26-0.47)
	Emergency referral + Prolonged or Obstructed labor	0.51 (0.37-0.66)
Antepartum hemorrhage	No emergency referral + No Antepartum hemorrhage	0.21 (0.15-0.25)
	Emergency referral + No Antepartum hemorrhage	0.23 (0.20-0.26)
	No emergency referral + Antepartum hemorrhage	0.35 (0.28-0.42)
	Emergency referral + Antepartum hemorrhage	0.36 (0.28-0.44)
Hypertensive disorders	No emergency referral + No Hypertensive disorders	0.23 (0.21-0.25)
	Emergency referral + No Hypertensive disorder	0.26 (0.21-0.31)
	No emergency referral + Hypertensive disorders	0.25 (0.20-0.31)
	Emergency referral + Hypertensive disorder	0.37 (0.31-0.43)

*A) Birthweight we adjusted for maternal employment, marital status, maternal education, parity, antepartum hemorrhage, prolonged or obstructed labor, hypertensive disorders, distance to facility, and emergency cesarean section. B) Prolonged or obstructed labor adjusted for maternal employment, marital status, maternal education, parity, antepartum hemorrhage, birthweight, hypertensive disorder, distance to facility, and emergency cesarean section. C) Antepartum hemorrhage adjusted for maternal employment, marital status, Maternal highest level of education, parity, prolonged or obstructed labor, birthweight, hypertensive disorders and distance to facility, emergency cesarean section. D) Hypertensive disorder adjusted for maternal employment, marital status, maternal education, parity, antepartum hemorrhage, birthweight, prolonged or obstructed labor, distance to facility, and emergency cesarean section

Supplementary Table 2: Predicted Probability of IP-NE by Obstetric Risk Groups and Emergency cesarean section Using Logistic Regression Margins

**Obstetric and fetal risk**		Probability (95%CI)
**Birthweight**	No CS + Normal birth weight	0.21 (0.18-0.23)
	CS + Normal birth weight	0.32(0.22-0.42)
	No CS + Low birth weight	0.32(0.21-0.42)
	CS + Low birth weight	0.39(0.32-0.47)
**Prolonged or Obstructed labor**	No CS + No Prolonged or Obstructed labor	0.21(0.19-0.23)
	CS + No Prolonged or Obstructed labor	0.31(0.23-0.40)
	No CS + Prolonged or Obstructed labor	0.73(0.51-0.95)
	CS + Prolonged or Obstructed labor	0.45(0.39-0.50)
**Antepartum hemorrhage**	No CS + No Antepartum hemorrhage	0.21(0.16-0.25)
	CS + No Antepartum hemorrhage	0.31(0.22-0.38)
	No CS + Antepartum hemorrhage	0.24(0.21-0.27)
	CS + Antepartum hemorrhage	0.37(0.23-0.51)
**Hypertensive disorders**	No CS + No Hypertensive disorders	0.23(0.10-0.36)
	CS + No Hypertensive disorder	0.25(0.10-0.40)
	No CS + Hypertensive disorders	0.24(0.21-0.26)
	CS + Hypertensive disorders	0.29(0.25-0.39)

*A) Birthweight adjusted for maternal employment, marital status, maternal education, parity, antepartum hemorrhage, prolonged or obstructed labor, hypertensive disorders, distance to facility, and emergency referral. B) Prolonged or obstructed labor adjusted for maternal employment, marital status, maternal education, parity, antepartum hemorrhage, birthweight, hypertensive disorder, distance to facility, and emergency referral. C) Antepartum hemorrhage adjusted for maternal employment, marital status, Maternal highest level of education, parity, prolonged or obstructed labor, birthweight, hypertensive disorders and distance to facility, and emergency referral. D) Hypertensive disorder adjusted for maternal employment, marital status, maternal education, parity, antepartum hemorrhage, birthweight, prolonged or obstructed labor, distance to facility, and emergency referral.
