# Supplementary material for: Prevalence and risk of adverse intrapartum-related outcomes in Uganda: a cross-sectional study with nested case–control
Source: BMJ Open. 2025 Oct 14;15(10):e099256. doi: 10.1136/bmjopen-2025-099256 (PMC12519689; doi:10.1136/bmjopen-2025-099256)
Supplement: Supplementary file 2 [file bmjopen-2025-099256.reviewer_comments.pdf]

PEER REVIEW HISTORY

BMJ Open publishes all reviews undertaken for accepted manuscripts. Reviewers are asked to complete a checklist review form and are provided with free text boxes to elaborate on their assessment. These free text comments are reproduced below.

ARTICLE DETAILS

Title (Provisional)

Prevalence and risk of adverse intrapartum-related outcomes in Uganda: a cross-sectional study with nested case-control

Authors

Wanduru, Phillip; Straneo, Manuela; Sadoo, Samantha; Tann, Cally J; Kakooza, Angelina Mwesige; Mutumba, Rolland; Annerstedt, Kristi Sidney; Waiswa, Peter; Hanson, Claudia

---

VERSION 1 - REVIEW

**Reviewer**	**1**
**Name**	**Kukka, Antti Juhani**
**Affiliation**	**Uppsala Universitet, Department of Women's and Children's Health**
**Date**	**01-Jun-2025**
**COI**	**None**

Thank you for the opportunity to review manuscript “Prevalence and risk of adverse intrapartum-related outcomes in Uganda: a cross-sectional study with nested case-control” submitted to the BMJ Open journal. The manuscript presents a novel way of looking at risk factors to key perinatal outcomes in the form of a nested case-control study. Previous papers have mostly presented case-control designs. As such, it is likely to generate interest for program planners trying to reduce intrapartum-related complications particularly in Sub-Saharan Africa. The apparent lack of protective effect from emergency referrals to reduce these complications will hopefully act as a catalyst for further studies. The manuscript at large is well written and has a red thread throughout from aim to methods, results and discussion.

I will offer my step-by-step comments below.

Abstract and key messages:

1. Clearly written, summarizes the contents well.
2. Row 48/49: "Adverse outcomes were higher among neonates whose mothers had antepartum hemorrhage (31.3%) and prolonged/obstructed labor (27.2%)." Space permitting, some specification on higher than what would be smart.

Research question and the introduction:

1. Aims are clearly stated, but please note that the first aim is written in current tense and second in past tense.
2. Statement "Globally, intrapartum-related neonatal encephalopathy (IP-NE) is the second leading cause of age-standardized disability-adjusted life years" misinterprets GBD data. Ref 3 is to GBD study of selected neurological conditions only. According to the 2021 GBD study, neonatal encephalopathy was 12th biggest cause to loss of age-adjusted DALYs overall (https://vizhub.healthdata.org/gbd-compare/).
3. In the second paragraph, how confident are you of the assertion that lack of quality of intrapartum care is the reason that intrapartum-related complications remain high despite increase in facility births? The explanation seems reasonable, but the references given do not address this exact question. Is any further proof available? If not, should the statement be even more cautious?
4. To me the key statement of the third paragraph is "there is limited evidence on how emergency interventions – such as referral and cesarean sections (CS) – modify the trajectory of these complications in SSA, a setting where delays are common" as it identifies a knowledge gap that the study is going to address. Did you find ANY previous publications on the question? Are there studies from other LMICs that could be lifted here? If not, perhaps worth highlighting that you are not aware of previous studies either here or in Strengths and Limitations.

Design, Methods and Participants:

1. I'm not enough familiar with nested case-control studies and the use of interaction terms in logistic regression to give a complete assessment of the statistical methods. Additional statistical review would be recommendable.
2. Methods do not mention a reporting guideline, but one is provided in the supplement.
3. It would be fair to clarify to the reader if the power calculation was done post-hoc as appears to be the case.

4. Study setting is clearly described but rather short. Following information would be relevant for the research question:
    a. Were emergency CS available 24/7? As you've mentioned the level of the NCUs (by the way an unnecessary abbreviation that is not repeated later on), it could have been smart to state the EmOC/BEmOC level too.
    b. Also please write something about the referral pathways to these hospitals as it's one of the key questions the manuscript is trying to elucidate. 7000 births per year for 4 million people is a very low number for Uganda so obviously most births take place somewhere else – where? Who gets to access these hospitals? For what cost?
5. Study population and variables
    a. While details ethical approvals are provided at the end of the manuscript, I found no mention of any form of informed consent procedure nor its absence in this manuscript. My understanding based on the description is that the study is essentially a registry-based exercise using routinely collected data, but even if no changes in routine care were done for the study, some ethical reflection would have been appreciated. The manuscript also lacks "Patient and public involvement" statement under methods that is requested by the BMJ Open journal.
    b. I find the section discussing variable definitions a little bit confusing to read. I understand that it's because of different outcomes in the cross-sectional and case-control studies, but would advise you to re-structure and clarify some details.
        i. On row 39/40 the IP-NE criteria are listed, and it would be helpful to properly clarify that all three were required already here as this info is provided only much later.
        ii. It's not clear to me how infants who received diagnosis of IP-NE but later died were classified in the study. Perhaps I just missed the explanation?
        iii. The cut-off for Thompson score was explained in detail, but the references 33 and 34 given to the lactate range suggest that the optimal cut-off to identify IR-NE is 7 to 9 mmol/l. Apgar <7 is also rather high number (e.g. 10.1016/j.ejogrb.2006.03.002). The cascading screening and requirement to fulfil all three criteria compensates for the permissive criteria though. See comment 2. Iii) in discussion.
        iv. What was the hospital policy for resuscitation of non-macerated stillbirths? Misclassification of stillbirths is a

huge issue across the world and decisions to resuscitate or not would impact all the three study outcomes.

v. Row 28-29 “to improve diagnostic precision, we adjusted categories as follows…”. Please clarify who are **we**? I see that there is a partial overlap between the authors of reference 38 and this manuscript.

vi. How was the presence or absence of extrauterine infections verified?

vii. The final paragraph on variables starting at the end of page six lists associated and confounding variables. No clear reasoning is provided why these variables were classified the way they were, but I see that something akin to Direct Acyclic Graphs are provided as supplementary material. Pleas refer to the Supplementary Figures in the text to clarify the reasoning!

Results:

1. Results are clearly presented and follow the research question and methods. Some minor stylistic comments:
   a. “Onethird (34.8%) of all births were primiparous mothers, while 15.6% were grand multipara (5+).” Perhaps specify 5+ deliveries?
   b. “Neonates with LBW were 12.6%, 83.3% were between normal birth weight, and 4.1% were high birthweight.” Would it not be easier to say that neonates **had** normal birth weight, **had** high birth weight?
   c. Row 21-22: Please clarify if neonatal mortality of 0.6% pertains to all births or live births? It appears to be 0,6% of all births but as neonatal mortality is typically presented as deaths/live births the matter needs specification.
   d. “We also observed that emergency referral was associated with an increased predicted probability of IP-NE within the obstetric and fetal risk groups; however, these associations did not reach statistical significance. For example, neonates whose mothers experienced prolonged or obstructed labor and were not referred had a predicted risk of 0.36 (95% CI: 0.26–0.47), which was higher but not statistically different from the risk of 0.51 (95% CI: 0.37–0.66) in those who were referred. **(Figure 2)”** Please reconsider how these statements are formulated. When a difference between two or more groups does not reach statistical significance, then the interpretation should be that there was no (statistically significant) difference. It’s potentially misleading to state that there was a difference, but it was not significant.

e. Final column in Table 2 has odd epitaph of “Born in poor condition” that I can’t remember seeing elsewhere in the manuscript. Is that necessary? Also n%(95%CI) as the column title seems erroneous as only %(95%CI) is reported. The abbreviation CI is missing from the table footnote.
f. Figure 3 seems to have been cut in the pdf and is lacking explanation to what each of the bars mean.

Discussion:

1. The opening paragraph does well to summarize the findings of the study and the following discussion adequately covers the merits and drawbacks of the manuscript. The findings are compared with previous studies and new references introduced as expected. The word notable is repeated twice during the opening section.
2. Additional alternative explanations to why emergency referral and emergency CS didn’t reduce the risk of IP-NE in other high-risk groups could be: i) type II error, particularly as your power calculation only gave 76% power for the main outcome. ii) these interventions might have resulted in survival of extremely sick fetuses that might have otherwise been classified as stillbirths. It would have been interesting to see similar analyses for stillbirths and deaths, but I understand that space is limited. iii) intrapartum hypoxia-ischemia is not the only reason why infants present with NE, some proportion of the infants would suffer from mimickers or a combination of risk factors that lie beyond the pathway of EmOC. Having relatively permissive diagnostic criteria for IP-NE might have slightly increased the proportion of infants who were misclassified as IP-NE.

Additional comments:

1) The numbering of supplementary figures and tables is not in order of appearance in the text and not all supplementary material are referenced in the text.
2) Row 21, Sub-Saharan Africa (SSA) is repetition from row 7/8. Please also note inconsistent spelling between these two occasions, and also the second bullet point in “What’s already known on this topic”.
3) References 7 and 8 are to Health Ministry U. Please check the formatting, I presume your reference manager needs to be told that the Ministry is not a person. Also ref 30 needs similar tweaking while ref 38 is incomplete.
4) Ref 13 is to a masters thesis. With plenty of peer reviewed publications supporting the relationship between maternal hypertension and intrapartum complications, I don’t see why one would cite a thesis. I agree with the authors

that studies from SSA are limited in number, but if the intention was to only site SSA papers, then 14 Butt, 16 Ellis and 18 Chen should not be included.

5) Ref 27 is to a StatPearls article. I don't see what additional information it provides to the other primary studies already cited
6) Introduction, Row 24/25: There are many ways to spell low-middle-income countries (LMICs) but most include and. Does BMJ offer guidance on "correct" spelling?
7) In methods, Row 29/30 – double punctuation "..19, 24"

Thank you for your attention and best of luck with your submission,

---

**Reviewer**	**2**
**Name**	**Dack, Kyle**
**Affiliation**	**University of Bristol, MRC Integrative Epidemiology Unit**
**Date**	**07-Jul-2025**
**COI**	**None**

Statistical review summary:

The methodology is reported in a transparent manner and the statistical analysis follows best practices. My primary suggestions are to add more information regarding how the margin analysis was performed, and to consider enhancements to the main results tables and figures.

Introduction:

1. Line 34. "There is limited evidence on how emergency interventions… in SSA". Does this mean there are few prior studies, or none? It would be beneficial to clarify either way, either to reference prior studies or to highlight that this is the first study of this topic.

2. I notice there is some inconsistency over which variables are the primary exposures/interventions of interest. The aims state that: "Second, we examined the association between IP-NE and i) emergency referrals and ii) emergency CS by obstetric and fetal risk factors."

However, in the methods section "independent variables" it is stated that the primary exposures are the obstetric and fetal risk factors, not emergency referrals and CS which are listed as explanatory factors (i.e. potential modifiers).

The statistical analysis section states that emergency referrals and CS are the interventions of interest, and make it sound as if the obstetric / fetal risk factors are just stratification factors or interaction terms.

Statistically it does not make much difference given that it is the interaction which is of interest. But it would help readers if these sections and any others were made consistent in terms of the labels used to describe the variables of interest.

Methods:

3. “A nested case-control study assessed the association between IP-NE, obstetric and fetal risk factors, emergency referral, and emergency CS.” This could be misread, as there is no clear distinction between the outcome, exposures, and modifying/interaction factors.

4. Study population: data extraction is described as ranging from June 1st to December 31st 2022. Was this range defined on a specific value such as date of admission, or does it encompass all recorded data. (e.g. both admission and outcome data must be within this date range).

5. The middle sections of the methodology make references to two different aspects of the study, “in the nested case control study”, “for the cross-sectional study”. The “study design” section introduced the nested case control study, but the cross-sectional study was not yet described – it would help readers to add a sentence to the study design section summarising it.

6. Outcome variables: was it possible for a child to be classified into multiple outcomes or are these mutually exclusive? In particular, outcomes B and C.

7. Independent variables: these are all described in good detail and it is clear to read. My only suggestion is to clarify the purpose of each group, i.e. whether they were measured to test the direct association, the interaction, as stratification factors, etc, to help clarify the distinction between the two sets of variables.

8. “We further applied post-estimation margins analysis to estimate the probability of IP-NE based on combinations of specific risk groups and interventions” could more details of this be added – e.g. which statistics was it based upon and how was it calculated. Was it applied to all 8 combinations of interventions and risk groups, or only selected ones?

Results

9. Table 1. “mode of birth” is nested under “emergency referral” which I suspect may be an error.

10. The statistical analysis section described interaction testing, which would be “further” followed by estimating predicted probabilities. The “further” implied to me

that both would be reported. However, the results only report the predicted probabilities. I suggest revising one of these sections to ensure consistency.

11. The predicted probabilities of the interactions are reported in bar charts, but it is quite hard to understand - the figures lack legends so I am not sure what the triangles or colours mean. The bar labels could also be refined “Emergency referral # Hypertensive disorder” – suggest replacing the hash sign with “&”.

12. Additionally, some readers may prefer a standard table, especially for quickly reviewing the results, and I think it would improve the clarity to report these results in both a table and figure format. In fact it looks like these are reported in supplementary tables, but these tables are not referenced in the text. It may be possible to compact these tables by spreading the first column along the horizontal axis, the authors may wish to explore this.

VERSION 1 - AUTHOR RESPONSE

**Reviewer 1**: Thank you very much for the insightful feedback, which has been invaluable in improving this manuscript.	
comment	Response
1. Line 34. “There is limited evidence on how emergency interventions… in SSA”.<br>Does this mean there are few prior studies, or none? It would be beneficial to<br>clarify either way, either to reference prior studies or to highlight that this is the first study of this topic.	Thank you for your observation. The original framing was unclear. We found no studies that assessed these interventions in their appropriate conceptual role as effect modifiers. Instead, they were often misclassified as risk factors—despite interventions being actions to mitigate risk, not sources of risk themselves. This misclassification can obscure the actual impact of timely interventions on modifying outcomes. We have revised the text accordingly.<br>**Line 98-111**<br>“In addition, there is a lack of evidence on the effectiveness of emergency interventions for preventing IP-NE from SSA. Risk factors for IP-NE such as hypertensive disorders,[15, 16] obstructed or prolonged labor,[13, 17, 18] antepartum hemorrhage,[13, 15] maternal infections,[19] and low birth weight (LBW) births,[16, 17] are well established in literature. However, the effectiveness of key emergency<br>interventions, including emergency CS and emergency referral, in modifying the association between these risk factors and IP-NE remains underexplored. Although widely recognised as critical strategies to reduce IP-NE, to our knowledge, no studies in SSA have examined whether these emergency interventions modify the association between these risk factors and IP-NE risk. Most analyses treat these emergency interventions as risk factors rather than effect modifiers, which can<br>misrepresent their role and limit understanding of their true

	impact in reducing IP- NE among high-risk births"
2. I notice there is some inconsistency over which variables are the primary exposures/interventions of interest. The aims state that: "Second, we examined the association between IP-NE and i) emergency referrals and ii) emergency CS by obstetric and fetal risk factors."<br>However, in the methods section "independent variables" it is stated that the primary exposures are the obstetric and fetal risk factors, not emergency referrals and CS which are listed as explanatory factors (i.e. potential modifiers). The statistical analysis section states that emergency referrals and CS are the interventions of interest, and make it sound as if the obstetric / fetal risk factors are just stratification factors or interaction terms. Statistically it does not make much difference given that it is the interaction which is of interest. But it would help readers if these sections and any others were made consistent in terms of the labels used to describe the variables of interest.	We acknowledge that the variable labels were confusing and could mislead readers. This issue has now been addressed throughout the entire document, including the abstract. For example, in the abstract, we mention<br>**Line 24-27**<br>"We assessed the prevalence of adverse intrapartum-related outcomes, evaluated the association between IP-NE and obstetric and fetal risk factors, and examined whether emergency referral and emergency caesarean section modified this association through interaction effects."<br>We have made sure to also add sub-headings to the variable description section
**Methods:**<br>3. "A nested case-control study assessed the association between IP-NE, obstetric and fetal risk factors, emergency referral, and emergency CS." This could be misread, as there is no clear distinction between the outcome, exposures, and modifying/interaction factors.	This is now edited as follows<br>**Line 124-127**<br>"A nested case-control study examined the association between IP-NE and obstetric and fetal risk factors, and assessed whether emergency referral and emergency CS modified this association through interaction effects."
4. Study population: data extraction is described as ranging from June 1st to December 31st 2022. Was this range defined on a specific value such as date of admission, or does it encompass all recorded data. (e.g. both admission and outcome data must be within this date range).	The births (and therefore outcomes) must have occurred during this period, regardless of the admission date.<br>**Line 149 to 151**<br>Edited as: "For the cross-sectional study, we extracted data of births that occurred between June 1, 2022 and , December 31, 2022, excluding neonates weighing less than 2,000 grams."
5. The middle sections of the methodology make references to two different aspects of the study, "in the nested case control study", "for the cross-sectional study". The "study design" section introduced the nested case control study, but the cross-sectional study was not yet described – it would help readers to add a sentence to the study design section summarising it.	Sorry that this was not clear, we now have tried to clarify<br>**Line 121 to 121**<br>" Our cross-sectional study we used prospectively collected data derived from the Action Leverage Evidence to Reduce Perinatal Mortality and Morbidity (ALERT) e- registry, [20] which included a 24-hour follow-up of all births to identify adverse perinatal outcomes.
6. Outcome variables: was it possible for a child to be classified into multiple outcomes or are these mutually exclusive? In particular, outcomes B and C.	Thank you very much for this question, which we appreciate. We agree that babies might have had different "outcomes" at different timepoints, but in our study, we used the outcome at 24h and defined it as mutually exclusive. Thus, if a baby

	survived 24 hours, it was either a healthy baby, a neonatal deaths or IP-NE
7. Independent variables: these are all described in good detail and it is clear to read. My only suggestion is to clarify the purpose of each group, i.e. whether they were measured to test the direct association, the interaction, as stratification factors, etc, to help clarify the distinction between the two sets of variables.	Thank you for this important observation. In the study variables description, we have added sub-heading to show outcome variables, independent variables, and then the effect modifiers.

8. "We further applied post-estimation margins analysis to estimate the probability of IP-NE based on combinations of specific risk groups and interventions" could more details of this be added – e.g. which statistics was it based upon and how was it calculated. Was it applied to all 8 combinations of interventions and risk groups, or only selected ones?	Thank you for this important questions. They have guided us to revise this section to make it more straight forward<br>**Line 226-242**<br><br>Further, we used multivariable logistic regression to assess whether emergency referral and emergency CS modified the association between obstetric and fetal risk factors and IP-NE. As shown in supplementary figure 2, each model included an interaction term between the presence or absence of intervention and a specific risk factor, adjusting for relevant confounders.<br><br>Effect modification was computed using post-estimation margins analysis, which calculates the predicted probability of IP-NE for all combinations of intervention status (present or absent) and risk factor categories. This method uses the regression coefficients—including interaction terms—to estimate adjusted probabilities reflecting how the intervention modifies the effect of each risk factor on IP-NE risk. The analysis was applied to all eight combinations arising from the two levels of each intervention crossed with the four key risk factors, allowing comprehensive assessment of modification effects across relevant strata.
**Results**<br>9. Table 1. "mode of birth" is nested under "emergency referral" which I suspect may be an error.	Thank you very much for spotting this and indeed this was a mistake which we corrected
10. The statistical analysis section described interaction testing, which would be "further" followed by estimating predicted probabilities. The "further" implied to me that both would be reported. However, the results only report the predicted probabilities. I suggest revising one of these sections to ensure consistency.	Thanks for the observation. We agree this was not very clear, so we revised it.<br>**Line 235-237**<br>"Effect modification was computed using post-estimation margins analysis, which calculates the predicted probability of IP-NE for all combinations of intervention status (present or absent) and risk factor categories."
11. The predicted probabilities of the interactions are reported in bar charts, but it is quite hard to understand - the figures lack legends so I am not sure what the triangles or colours mean. The bar labels	Thank you for your observation. The graphs have been revised entirely

could also be refined "Emergency referral # Hypertensive disorder" – suggest replacing the hash sign with "&".	
12. Additionally, some readers may prefer a standard table, especially for quickly reviewing the results, and I think it would improve the clarity to report these	Thank you for this observation, we now have the tables, supplementary tables 1 and 2 referenced in the results section

results in both a table and figure format. In fact it looks like these are reported in supplementary tables, but these tables are not referenced in the text. It may be possible to compact these tables by spreading the first column along the horizontal axis, the authors may wish to explore this	
REVIEWER 2: I greatly appreciate your thoughtful feedback, which has been instrumental in strengthening this manuscript	
Abstract and key messages:<br>1. Clearly written, summarizes the contents well.<br>2. Row 48/49: "Adverse outcomes were higher among neonates whose mothers had antepartum hemorrhage (31.3%) and prolonged/obstructed labor (27.2%)." Space permitting, some specification on higher than what would be smart	Thank you for this observation and we revised.<br>**Line 47-49**<br>Adverse outcomes were higher among neonates whose mothers had antepartum haemorrhage (31.3%) or prolonged/obstructed labour (27.2%) compared to those whose mothers had no complications.
Research question and the introduction:<br>1. Aims are clearly stated, but please note that the first aim is written in current tense and second in past tense	It has been revised accordingly to make it past tense
2. Statement "Globally, intrapartum-related neonatal encephalopathy (IP-NE) is the second leading cause of age-standardized disability-adjusted life years" misinterprets GBD data. Ref 3 is to GBD study of selected neurological conditions only. According to the 2021 GBD study, neonatal encephalopathy was 12th biggest cause to loss of age-adjusted DALYs overall (https://vizhub.healthdata.org/gbd-compare/)	Thank you for this important comment. we have clarified that it is the second leading among neurological disorders and updated the reference<br>**Line 78-79**<br>"Globally, intrapartum-related neonatal encephalopathy (IP-NE) is the second leading cause of age-standardised disability-adjusted life years among neurological disorders.[3]"

3. In the second paragraph, how confident are you of the assertion that lack of quality of intrapartum care is the reason that intrapartum-related complications remain high despite increase in facility births? The explanation seems reasonable, but the references given do not address this exact question. Is any further proof available? If not, should the statement be even more cautious?	Thank you for the valuable observation. The WHO, through its Quality of Care initiative, has stated that inadequate quality of care at birth is a key reason why intrapartum-related complications persist despite increased coverage of facility births. We have edited the paragraph accordingly and referenced relevant WHO documents<br>**Line 85-91**<br>Efforts to prevent intrapartum-related complications have focused on facility births, and major increases in these have been seen in the last decade; in Uganda, over 90% of births now occur in facilities.[4] However, this shift has not been paralleled by a corresponding reduction in adverse perinatal outcomes, which the WHO largely<br>attributes to inadequate quality of intrapartum care.[5, 6] In response, WHO guidelines

	and key Ugandan policies prioritise improving the quality of care in health facilities.<br>5, 7, 8
4. To me the key statement of the third paragraph is "there is limited evidence on how emergency interventions – such as referral and cesarean sections (CS) – modify the trajectory of these complications in SSA, a setting where delays are common" as it identifies a knowledge gap that the study is going to address. Did you find ANY previous publications on the question? Are there studies from other LMICs that could be lifted here? If not, perhaps worth highlighting that you are not aware of previous studies either here or in Strengths and Limitations	Thank you for the observation, which was also highlighted by reviewer 1. The gap is that we found no studies assessing these interventions as effect modifiers—their proper conceptual role; most analyses misclassified them as risk factors. We have revised the text accordingly.<br>**Line 98-111**<br>"In addition, there is a lack of evidence on the effectiveness of emergency interventions for preventing IP-NE from SSA. Risk factors for IP-NE such as hypertensive disorders,[15, 16] obstructed or prolonged labor,[13, 17, 18] antepartum hemorrhage,[13, 15] maternal infections,[19] and low birth weight (LBW) births,[16, 17] are well established in literature. However, the effectiveness of key emergency interventions, including emergency CS and emergency referral, in modifying the association between these risk factors and IP-NE remains underexplored. Although widely recognised as critical strategies to reduce IP-NE, to our knowledge, no studies in SSA have examined whether these emergency interventions modify the association between these risk factors and IP-NE risk. Most analyses treat these<br>emergency interventions as risk factors rather than effect modifiers, which can<br>misrepresent their role and limit understanding of their true impact in reducing IP- NE among high-risk births"

**Design, Methods and Participants:**<br>1. I’m not enough familiar with nested case-control studies and the use of interaction terms in logistic regression to give a complete assessment of the statistical methods. Additional statistical review would be recommendable.	Thank you for this. We have carefully ensured statistical appropriateness and robustness, and we have conducted consultations.
2. Methods do not mention a reporting guideline, but one is provided in the supplement.	The reporting guideline was a journal prerequisite, which we completed as part of the submission forms. Because it was already included in these materials, we did not consider it necessary to restate it in the Methods section
3. It would be fair to clarify to the reader if the power calculation was done post- hoc as appears to be the case.	Thanks, we have added that it was done post-hoc.<br>Our power calculation, conducted post-hoc, confirmed that the case-control study was adequately powered (Supplementary Figure 1).
4. Study setting is clearly described but rather short. Following information would be relevant for the research question:<br>a. Were emergency CS available 24/7? As you’ve mentioned the level of	We have adjusted the study setting to add components highlighted by the reviewer.<br>However, we did not do an assessment using the BEMOC criteria, which is why we would like to abstain from using this definition.
the NCUs (by the way an unnecessary abbreviation that is not repeated later on), it could have been smart to state the EmOC/BEmOC level too.<br>b. Also please write something about the referral pathways to these hospitals as it’s one of the key questions the manuscript is trying to elucidate. 7000 births per year for 4 million people is a very low number for Uganda so obviously most births take place somewhere else – where? Who gets to access these hospitals? For what cost?	**Line 128-145**<br>The study was conducted in a Regional Referral Hospital and a General Hospital in Eastern Uganda, serving a predominantly rural population of about 4 million. Uganda has 14 regions, each with a tiered referral health system, with Regional Referral Hospitals at the highest level, followed by General Hospitals that typically serve multiple districts. Health Centre IVs and IIIs also provide maternity care and handle most uncomplicated obstetric cases within these districts. Both hospital types provide maternity care—including cesarean sections, neonatal care, blood transfusions, and laboratory services. Neonatal care units at these facilities offer WHO Level 2 care. [21] Regional Referral Hospitals are staffed by consultant obstetricians and pediatricians, while General Hospitals have non-consultant specialists. Caesarean sections are available 24/7, though delays occur due to stockouts and staf f shortages. These public hospitals offer free services and handle high birth volumes, with approximately 7,000 deliveries annually each. Despite service availability, staffing remains limited, and several studies have documented quality-of-care gaps.[20, 22-24]

5. Study population and variables<br>a. While details ethical approvals are provided at the end of the manuscript, I found no mention of any form of informed consent procedure nor its absence in this manuscript. My understanding based on the description is that the study is essentially a registry-based exercise using routinely collected data, but even if no changes in routine care were done for the study, some ethical reflection would have been appreciated. The manuscript also lacks "Patient and public<br>involvement" statement under methods that is requested by the BMJ Open journal.	Thank you very much for your valuable observation. Although we did not describe the informed consent procedure in detail within the manuscript, it is implied through the ethical approval certificates cited in the ethics section. All participants provided informed consent in accordance with the country's regulatory requirements.<br>**Line 424-427**<br>"This study received ethical clearance from the Higher Degrees Research and Ethics Committee of Makerere University School of Public Health (SPH-2021-126), the Uganda National Council of Science and Technology (HS2057ES), and National Ethics<br>Approval body in Sweden (Etikprövningsmyndigheten Dnr 2022-04563-01)"
b. I find the section discussing variable definitions a little bit confusing to read. I understand that it's because of different outcomes in the cross-<br>sectional and case-control studies, but would advise you to re-structure<br>and clarify some details.	We have made efforts to make clear which are the outcome variables, risk factors and effect modifiers

i. On row 39/40 the IP-NE criteria are listed, and it would be helpful to properly clarify that all three were required already here as this info is provided only much later.	Done and now it reads as<br>**Line 157-158**<br>"….cases were neonates with IP-NE defined by Apgar <7 at 5 minutes, and cord blood lactate ≥ 5.5 mmol/l, and a Thompson score ≥5."
ii. It's not clear to me how infants who received diagnosis of IP-NE but later died were classified in the study.<br>Perhaps I just missed<br>the explanation?	We limited follow-up to 24 hours, which represents the full extent of our knowledge.
iii. The cut-off for Thompson score was explained in detail, but the references 33 and 34 given to the lactate range suggest that the optimal cut-off to identify IR-NE is 7 to 9 mmol/l. Apgar <7 is also rather high number (e.g. 10.1016/j.ejogrb.2006.03.002). The cascading screening and requirement to fulfil all three criteria compensates for the permissive criteria though. See comment 2. Iii) in discussion.	Thank you for the insightful comment. We acknowledge that the use of an Apgar score <7 as a cutoff point is high; however, this was based on the WHO-recommended threshold for defining a non-reassuring Apgar score at 5 minutes. Indeed, as you mention, we compensate by using three criteria.<br>For lactate cut off, we have now updated the reference for the optimal umbilical cord lactate cutoff to cite a recent Ugandan study:<br>Ayebare E, Tumwine JK, Nankunda J, Hjelmstedt A, Jonas W, Ndeezi G, et al.<br>Evaluating predictive values of umbilical cord arterial lactate for adverse newborn outcomes among term-births in northern Uganda: A cross sectional analytical study. Int J Gynaecol Obstet. 2025;169(1):408-20. Epub 20241130. doi:<br>10.1002/ijgo.16037. PubMed PMID: 39614695; PubMed Central PMCID: PMC11911978.

iv. What was the hospital policy for resuscitation of non-macerated stillbirths? Misclassification of stillbirths is a huge issue across the world and decisions to resuscitate or not would impact all the three study outcomes.	Thank you for your comment. Currently, there is no hospital policy regarding the resuscitation of non-macerated stillbirths. While this is an important topic, it falls outside the scope of our work, so we do not address it.
v. Row 28-29 "to improve diagnostic precision, we adjusted categories as follows…". Please clarify who are we? I see that there is a partial overlap between the authors of reference 38 and this manuscript.	This was an error; the references were not intended to be included. Our team decided to adjust the cutoff. The issue has now been corrected.
vi. How was the presence or absence of extrauterine infections verified?	We based our findings on the patient chart documentation. Now, a statement has been added<br>**Line 194**

	"<u>extra-uterine infections, which were defined as the presence of HIV, syphilis, or malaria as documented in the patient charts"</u>
vii. The final paragraph on variables starting at the end of page six lists associated and confounding variables. No clear reasoning is provided why these variables were classified the way they were, but I see that something akin to Direct Acyclic Graphs are provided as supplementary material. Pleas refer to the Supplementary Figures in the text to clarify the reasoning!	Thank you for the comment. We believe this confusion may be because variables were not labelled clearly: that is, risk factors, outcome factors, and effect modifiers. We have now addressed these and believe they resolve the issue.
Results are clearly presented and follow the research question and methods. Some minor stylistic comments:<br>a. "Onethird (34.8%) of all births were primiparous mothers, while 15.6% were grand multipara (5+)." Perhaps specify 5+ deliveries?	Thank you for the positive feedback. We have addressed the comment on parity. This has been revised accordingly to indicate multiparous are 5+ deliveries
b. "Neonates with LBW were 12.6%, 83.3% were between normal birth weight, and 4.1% were high birthweight." Would it not be easier to say that neonates had normal birth weight, had high birth weight?	Neonates with high birth weight are not the same as those with normal weight; they have a known high risk of adverse perinatal outcomes. We just wanted to highlight this percentage
c. Row 21-22: Please clarify if neonatal mortality of 0.6% pertains to all births or live births? It appears to be 0,6% of all births but as neonatal mortality is typically presented as deaths/live births the matter needs specification.	It was all the births, and this clarification has been added<br>**Line 274**<br>"<u>Neonatal deaths within 24 hours were 0.6% of all births"</u>

d. “We also observed that emergency referral was associated with an<br>increased predicted probability of IP-NE within the obstetric and fetal risk groups; however, these associations did not reach statistical significance. For example, neonates whose mothers experienced prolonged or obstructed labor and were not referred had a predicted risk of 0.36 (95% CI: 0.26–0.47), which was higher but not statistically<br>different from the risk of 0.51 (95% CI: 0.37–0.66) in those who were referred. (Figure 2)”<br>Please reconsider how these statements are formulated. When a difference between two or more groups does not reach statistical significance, then the interpretation should be that there was no (statistically significant) difference. It’s potentially misleading to state that there was a difference, but it was not significant.	We acknowledge that the observed associations did not reach statistical<br>significance, as clearly indicated in the manuscript. Nevertheless, reporting the direction of these associations is important, as it provides valuable insight into potential trends. Statistical significance alone does not fully capture the relevance of findings, especially in studies with limited sample sizes such as ours. Therefore, presenting the direction of effects supports a more nuanced interpretation and may help inform future research hypotheses.
e. Final column in Table 2 has odd epitaph of “Born in poor condition” that<br>I can’t remember seeing elsewhere in the manuscript. Is that necessary?	Born in poor condition has been deleted.
Also n%(95%CI) as the column title seems erroneous as only %(95%CI) is reported. The abbreviation CI is missing from the table footnote.<br>f. Figure 3 seems to have been cut in the pdf and is lacking explanation to what each of the bars mean.	
**Discussion:**<br>1. The opening paragraph does well to summarize the findings of the study and the following discussion adequately covers the merits and drawbacks of the manuscript. The findings are compared with previous studies and new references introduced as expected. The word notable is repeated twice during the opening section.	Thank you for this comment, I have deleted one of the notable.

2. Additional alternative explanations to why emergency referral and emergency CS didn't reduce the risk of IP-NE in other high-risk groups could be:<br>i) type II error, particularly as your power calculation only gave 76% power for the main outcome.<br>ii) these interventions might have resulted in survival of extremely sick fetuses that might have otherwise been classified as stillbirths. It would have been interesting to see similar analyses for stillbirths and deaths, but I understand that space is limited.<br>iii) intrapartum hypoxia-ischemia is not the only reason why infants present with NE, some proportion of the infants would suffer from mimickers or a combination of risk factors that lie beyond the pathway of EmOC. Having relatively permissive diagnostic criteria for IP-NE might have slightly increased the proportion of infants who were misclassified as IP-NE	Thanks for the careful observation.<br>**Line 3740378**<br>i) Now we have added a limitation about not making 80% power benchmark: Further, the statistical power for the nested case-control study assessing the modifying effects of emergency referral and emergency cesarean section was 76%. While this is reasonably close to the conventional 80% benchmark—suggesting adequate ability to detect the main effects—it still carries a modest risk of a Type II error.<br>ii) We agree that investigating the modifying effect on stillbirths would be beneficial; however, it is beyond the scope of this work.<br>iii) We trust that our diagnostic criteria for IP-NE were both stringent and reliable, as they combined Apgar score, lactate measurement, and the validated Thompson score. While we recognise that neonatal encephalopathy can sometimes be influenced or exacerbated by factors other than hypoxic ischemia, we believe that hypoxic ischemia was the predominant cause in the majority of cases included in our study.
Additional comments:<br>1) The numbering of supplementary figures and tables is not in order of appearance in the text and not all supplementary material are referenced in the text.<br>2) Row 21, Sub-Saharan Africa (SSA) is repetition from row 7/8. Please also note	We have fixed the error of numbering supplementary figures, proofread the document to ensure repetitions are removed, and fixed the references as advised.

inconsistent spelling between these two occasions, and also the second bullet point in "What's already known on this topic".<br>3) References 7 and 8 are to Health Ministry U. Please check the formatting, I presume your reference manager needs to be told that the Ministry is not a person.<br>Also ref 30 needs similar tweaking while ref 38 is incomplete.<br>4) Ref 13 is to a masters thesis. With plenty of peer reviewed publications supporting the relationship between maternal hypertension and intrapartum complications, I don't see why one would cite a thesis. I agree with the authors that studies from SSA are limited in number, but if the intention was to only site SSA papers, then 14 Butt, 16 Ellis and 18	

Chen should not be included.	

VERSION 2 - REVIEW

Reviewer 1

Name Kukka, Antti Juhani

Affiliation Uppsala Universitet, Department of Women's and Children's Health

Date 31-Aug-2025

COI

Thank you for addressing my feedback in a thorough manner. I find that the edits made were sufficient to address my concerns, and adequate reasoning was given for leaving out some of the suggestions. I have one remaining query and three smaller notes.

1. I feel that my question 5bii, which is the same as question 6 posed by Reviewer 1, was not adequately addressed. The first paragraph under headline Outcomes should specify that the outcomes were mutually exclusive and that infants who had IP-NE but died within 24 hours were classified as neonatal deaths as explained in the response to reviewer 1.

Secondly, as early mortality in IP-NE can be in the excess of 20%, a large proportion of the infants who were dead at 24 hours might have had IP-NE prior to death. The second part of the study examines whether emergency interventions modify the association between selected obstetric risk factors and IP-NE. It would have thus been prudent to include even those infants who had IP-NE at 6-7 and/or 12-13 hours but died before 24 hours as cases. The way I read the current methods, it was actually "infants with IP-NE who survived alive until at least 24 hours of life", that were in the case group. Please clarify the case definition for the case-control study.

2. Row 195 should read "IP-NE defined as below." (rather than above)

3. Reference 12 seems to be missing from the edited text as line 107 was deleted. Please check that the referencing runs correctly.

4. Row 375 introduces an abbreviation APH that has not been used in the text.

Thank you for your attention and best of luck with your submission!

VERSION 2 - AUTHOR RESPONSE

**Comments from reviewer:**<br>Thank you for addressing my feedback in a thorough manner. I find that the edits made were sufficient to address my concerns, and adequate reasoning was given for leaving out some of the suggestions. I have one remaining query and three smaller notes.	We thank the reviewer for dedicating time and giving us constructive feedback.
A. I feel that my question 5bii, the same as question 6 posed by Reviewer 1, was not adequately addressed. The first paragraph under the headline Outcomes should specify that the outcomes were mutually exclusive and that infants who had IP-NE but died within 24 hours were classified as neonatal deaths, as explained in the response to Reviewer 1.<br>B. Secondly, as early mortality in IP-NE can be in the excess of 20%, a large proportion of the infants who were dead at 24 hours might have had IP-NE prior to death. The second part of the study examines whether emergency interventions modify the association between selected obstetric risk factors and IP-NE. It would have thus been prudent to include even those infants who had IP-NE at 6-7 and/or 12-13 hours but died before 24 hours as cases. The way I read the current methods, it was actually "infants with IP-NE who survived alive until at least 24 hours of life", that were in the case group. Please clarify the case definition for the case-control study	Thank you for this observation. Throughout the methods section, we have made it clear that IP-NE were those that survived 24 hours.<br>**Lines 218 to 228**<br>For the cross-sectional study, we defined three mutually exclusive primary outcomes, including (a) Stillbirths, defined as a foetus delivered without signs of life, as observed and documented by health workers present at birth. If evidence of maceration was present, they were classified as 'antepartum-related stillbirth'; those without evidence of maceration were classified as 'intrapartum-related stillbirths'; (b) neonatal deaths, defined as death of a neonate within 24 hours of birth regardless whether signs and symptoms of IP-NE were observed ; (c) IP-NE neonates who met the criteria for IP-NE and were alive at 24 hours (see definition below).<br>In the case–control study, the primary outcome of interest was IP-NE among neonates alive at 24 hours.
2. Row 195 should read "IP-NE defined as below." (rather than above)	This has been done on line **226**
3. Reference 12 seems to be missing from the edited text as line 107 was deleted. Please check that the referencing runs correctly.	The reference is there, and it is updated.
4. Row 375 introduces an abbreviation APH that has not been used in the text.	We have written it in full.

VERSION 3 - REVIEW

Reviewer 1

Name Kukka, Antti Juhani

Affiliation **Uppsala Universitet, Department of Women's and Children's Health**

Date **28-Sep-2025**

COI

Thank you for addressing my questions. I have no further comments. Best of luck with your submission!
